# Pioglitazone Ameliorates Renal Ischemia-Reperfusion Injury **via** Inhibition of NF-κB Activation and Inflammation in Rats

**DOI:** 10.3389/fphys.2021.707344

**Published:** 2021-07-19

**Authors:** Gaode Zou, Zhiyu Zhou, Xiaoqing Xi, Ruizhen Huang, Honglin Hu

**Affiliations:** ^1^Department of Urology, The Second Affiliated Hospital of Nanchang University, Nanchang, China; ^2^Department of Pathology, College of Basic Medicine, Jiangxi University of Traditional Chinese Medicine, Nanchang, China

**Keywords:** pioglitazone, renal ischemia-reperfusion injury, NF-κB signaling pathway, inflammation, protective effect

## Abstract

Renal ischemia-reperfusion injury (IRI) is considered as a major cause of acute kidney injury. In this study, we investigated the role of the NF-κB signaling pathway and inflammation in the amelioration of renal IRI using pioglitazone. Sprague–Dawley (SD) rats were subjected to bilateral renal artery clamping for 45 min followed by perfusion restoration for establishing a simulated renal IRI model. At 24 h post-operatively, we assessed the serum levels of creatinine and urea nitrogen, expression levels of peroxisome proliferator-activated receptor gamma (PPAR-γ) and NF-κB-related (p-IKK-β and IκB-α) proteins, and mRNA expression levels of the inflammatory cytokines, including TNF-α and MCP-1, in the renal tissue of various study groups. The histopathological evaluation of renal tissue was also conducted. In rat renal tissue, pioglitazone treatment decreased the serum levels of post-renal IRI creatinine and urea nitrogen, as well as necrosis. Furthermore, it elevated the expression of PPAR-γ protein and decreased the expression of NF-κB-related proteins. Pioglitazone also decreased the mRNA expression of TNF-α and MCP-1 in the renal tissue. Thus, pioglitazone ameliorates renal IRI by inhibiting the NF-κB signaling pathway and inflammatory response in rats.

## Introduction

Renal ischemia-reperfusion injury (IRI) is a common pathophysiological phenomenon in clinical settings. It is considered as a major cause of acute renal failure and the main factor in early recovery of renal graft function and long-term survival post renal transplantation. The pathological mechanism of IRI is complex and involves many biological processes. Most importantly, it is believed that an increase in free radical production, intracellular calcium overload, and excessive activation of inflammatory response are cumulatively responsible for IRI. The microvascular and parenchymal organ damage induced upon ischemia tissue reperfusion is mainly attributed to the reactive oxygen-free radicals, and it has been demonstrated in many organs. The production of antioxidant enzymes that scavenge free radicals in ischemic tissue is then impaired, thereby exacerbating the damage caused by these free radicals in the post ischemic reperfusion tissue. However, free radical scavenging by superoxide dismutase (SOD) protects against IRI (Huang et al., 2021; Ostojic et al., 2021).

Pioglitazone hydrochloride, an insulin-sensitizing agent and thiazolidinedione, has previously been described as a synthetic ligand of peroxisome proliferator-activated receptor-gamma (PPAR-γ), and it has been shown to activate PPAR-γ by up-regulating extracellular signal-regulated kinases and cyclooxygenase-2, suppressing NLRP3 inflammasome activities, and modulating PPAR-γ and heme-oxygenase 1, thereby ameliorating IRI in organs, such as the myocardium, brain, retina, and ovary (Kaundal et al., 2009; Wang et al., 2012; Zhang et al., 2017; Refaie and El-Hussieny, 2018). We previously demonstrated that pioglitazone improves renal IRI by inhibiting apoptosis in renal tubular epithelial cells, alleviating oxidative stress, and enhancing autophagy (Hu et al., 2012; Zou et al., 2013; Chen et al., 2018). Therefore, in this study, we aimed to investigate the molecular mechanisms underlying pioglitazone hydrochloride-mediated amelioration of renal IRI with respect to the nuclear factor-kappaB (NF-κB) signaling pathway and inflammatory response using a rat model.

## Materials and Methods

### Experimental Animals

Specific pathogen-free male Sprague–Dawley (SD) rats, weighing 200–250 g were purchased from the Laboratory Animal Center of Nanchang University (Nanchang, Jiangxi, China). The animals had *ad libitum* access to food and water.

### Ethical Considerations

This study was approved by the Ethics Committee for Animal Experiments of the Second Affiliated Hospital of Nanchang University, China (No.20140312002).

### Experimental Reagents

Protein extraction and purification kits, along with rabbit anti-rat peroxisome proliferator-activated receptor-gamma (anti-PPAR-γ), IκB kinase beta phosphorylation (anti-p-IKK-β), and inhibitor κB-alfa (IκB-α) antibodies were purchased from Wuhan Boster Biological Technology, Ltd., (Hubei, China). Horseradish peroxidase (HRP) conjugated secondary antibody was purchased from TRANS (United States). Enhanced Chemi-Luminescence (ECL) immunodetection kit was purchased from Thermo Fisher Scientific (United States).

### Grouping of Experimental Animals

The male SD rats were randomly assigned to the following groups: (1) Renal IRI group (*n* = 6); (2) Pioglitazone (Pio) + Renal IRI group (*n* = 6); (3) GW9662 (PPAR-γ antagonist) + Pio + Renal IRI group (*n* = 6); (4) GW9662 + Renal IRI group (*n* = 6); (5) Pio + Sham operation group (*n* = 6); (6) GW9662+ Sham operation group (*n* = 6); and (7) Sham operation group (*n* = 6). Rats in the Pio groups were administered pioglitazone for 3 days before the surgery (10 mg/kg body weight once a day *via* intraperitoneal injection), and the GW9662 group was administered GW9662 once at 4 h before surgery (1 mg/kg body weight once a day *via* intraperitoneal injection) (Chen et al., 2018).

### Rat Model of Renal IRI

Rats were abstained for 8–12 h before surgery. Thereafter, they were anesthetized intraperitoneally by administering 2% chloral hydrate at a volume of 2 mL/100 g body weight. The surgical process was as follows: A 1.5–2 cm midline incision was made on the abdomen, and the abdominal wall was separated layer-wise to enter into the abdominal cavity. The intestine was pushed aside to expose the bilateral renal pedicles, which were fastened for 45 min using atraumatic clamps, occlusion was verified visually based on the change in the color of the kidneys to a lighter shade and reperfusion based on a blush color. Restoration of renal blood flow was achieved upon removing the clamps, and then, the incision was closed. Saline (0.9%) was injected intraoperatively to ensure adequate hydration of the experimental animals. After the surgery, the animals had *ad libitum* access to food and water. The animals in the sham operation group were subjected to the same surgical procedure except for the fastening of the bilateral renal pedicles.

### Measurement of Plasma Glucose Level

The animals in Pio groups were administered pioglitazone for 3 days (10 mg/kg body weight once a day *via* intraperitoneal injection), and those in the control group were fed normal diets for 3 days. Blood samples were collected from the venous plexus at the medial canthal region at two different time points, before and 1 h after feed in the morning. The rats were abstained from day 3 in the evening until day 4 early in the morning, and then, they were allowed to feed. The blood samples were taken on day 4 in the morning before and 1 h after restarting the feed. The samples were immediately collected into heparinized ice-cold centrifuge tubes and stored at −80°C until the assays were performed to assess the plasma glucose levels.

### Assessment of Renal Function

About 24 h postoperatively, 0.5 mL of blood was collected from the venous plexus at the medial canthal region and centrifuged at 4,500 rpm for 10 min. Serum was collected to assess the levels of creatinine and urea nitrogen.

### Histopathological Evaluation of Renal Tissue

Rats in all groups were euthanized through spinal dislocation 24 h postoperatively. The kidneys were removed from each rat, dissected along the major axis, fixed using 10% neutral buffered formalin, embedded in paraffin, and sectioned into 4 μm slices for hematoxylin and eosin (H&E) staining. Necrosis in the renal tubules was observed using the optical microscope. For each slide, 20 fields of view at the corticomedullary junction were randomly selected for semi-quantitative evaluation of the degree of renal tubular necrosis according to the method described by Rabb et al. (1994). Higher score indicated higher degree of necrosis (maximum score = 4), 0 = normal kidney; 1 = minimal necrosis, <5% involvement; 2 = mild necrosis, 5–25% involvement; 3 = moderate necrosis, 25–75% involvement; and 4 = severe, >75% involvement.

### Reverse Transcription Polymerase Chain Reaction

The kidneys were collected 24 h postoperatively, homogenized on ice, and centrifuged. A certain amount of renal tissue was harvested and lysed in TRIzol according to the manufacturer’s instructions, and total RNA was extracted using the phenol-chloroform method. Reverse transcription polymerase chain reaction (RT-PCR) was performed using the synthesized cDNA as template to evaluate the mRNA expression of PPAR-γ, U6 (the internal reference gene), tumor necrosis factor-α (TNF-α), and monocyte chemotactic protein-1 (MCP-1). The cDNA samples were stored at −20°C. Quantitative PCR (qPCR) was performed for 40 cycles on an ABI PRISM 7300 Real-time PCR System using the reaction conditions according to the manufacturer’s instructions. A 20-μL reaction mixture was used, in which 2 μL of the reverse transcription products was separately reacted with the specific primers of PPAR-γ, TNF-α, MCP-1, and U6. The expression data were analyzed using the 2^–ΔΔ*CT*^ method (Chen et al., 2018).

### Western Blotting

The protein concentrations were evaluated using the Coomassie Brilliant Blue (Bradford) assay. The protein samples were resolved through SDS-PAGE using a 10% SDS-polyacrylamide gel, following which the resolved protein bands were transferred to a nitrocellulose membrane. After blocking the nitrocellulose membrane overnight using 5% non-fat milk prepared in tris buffered saline tween (TBST), rabbit anti-rat PPAR-γ, rabbit anti-rat p-IKK-β, and rabbit anti-rat IκB-α antibodies (1:400) were separately added for incubation at 4°C overnight. Subsequently, the corresponding HRP-conjugated secondary antibody was added. Color development, exposure, and X-ray film development were performed using the ECL immunodetection kit according to the manufacturer’s instructions. After scanning, the grayscale values of the bands were measured using the Image-Pro Plus software. β-actin was used as the loading control (Chen et al., 2018).

### Statistical Analysis

The experimental data were statistically analyzed and processed using SPSS 22.0. The quantitative data were expressed as mean (x) ± S.E. The mean values of two groups were compared using the *t*-test, and multiple means were compared using the one-way ANOVA along with the Tukey *post hoc* multiple-comparisons test. The results were considered statistically significant when *p* < 0.05.

## Results

### Pioglitazone Treatment Did Not Affect Plasma Glucose Levels in Rats

**FIGURE 1:**
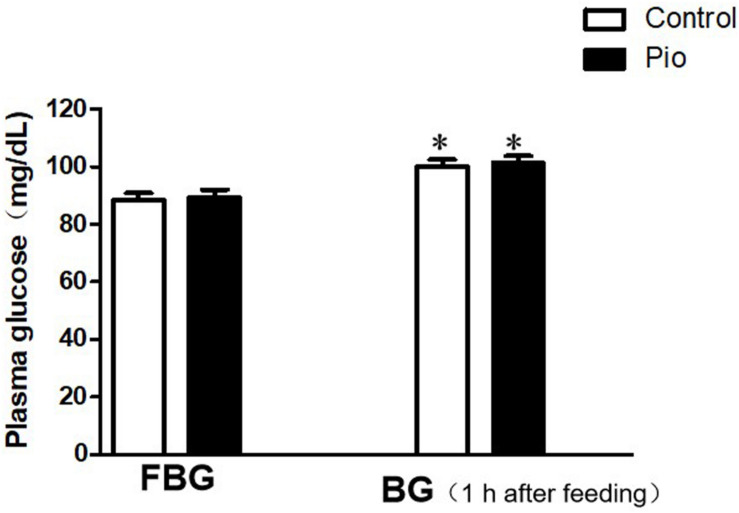
Comparison of the plasma glucose levels before and 1 h after feeding. There were no significant differences in the plasma glucose levels before and 1 h after feeding between the control and pioglitazone-treatment group. Values are expressed as mean ± SE (*n* = 6). (FBG, fasting blood glucose; BG, blood glucose). Compared to FBG, ^∗^ represents *p* < 0.05.

The comparison of the plasma glucose levels is presented in Figure 1. Although the blood glucose level increased significantly at 1 h after feeding compared to that before feeding in both groups, there were no significant differences in the blood glucose levels before and 1 h after feeding, respectively, between the control and pioglitazone treatment group.

### Pioglitazone Treatment Decreased Serum Creatinine and Urea Nitrogen Levels in Rats With Renal IRI

**FIGURE 2:**
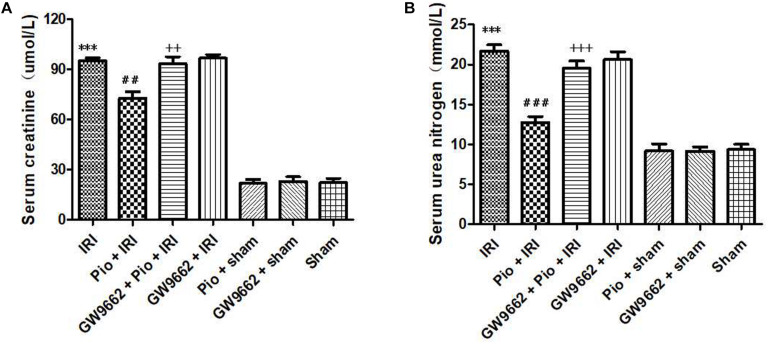
Pioglitazone treatment improves renal IRI. **(A)** Serum creatinine levels in different groups at 24 h after renal IRI. **(B)** Serum urea nitrogen levels in different groups at 24 h after renal IRI. Values are expressed as mean ± SE (*n* = 6). Compared to the sham group, ^∗∗∗^ represents *p* < 0.001; compared to the IRI group, ## represents *p* < 0.01, and ### represents *p* < 0.001; compared to the Pio + IRI group, ++ represents *p* < 0.01, and +++ represents *p* < 0.001.

At 24 h postoperatively, the renal IRI group exhibited significantly higher serum creatinine and urea nitrogen levels than that in the sham operation group. However, the increase in the serum creatinine and urea nitrogen levels was significantly suppressed in renal IRI rats that were treated with pioglitazone. When pioglitazone was used in combination with the PPAR-γ antagonist, GW9662, the renal protective effects of pioglitazone were repressed (Figure 2).

### Pioglitazone Treatment Alleviated Renal Tubular Injury in Rats With Renal IRI

**FIGURE 3:**
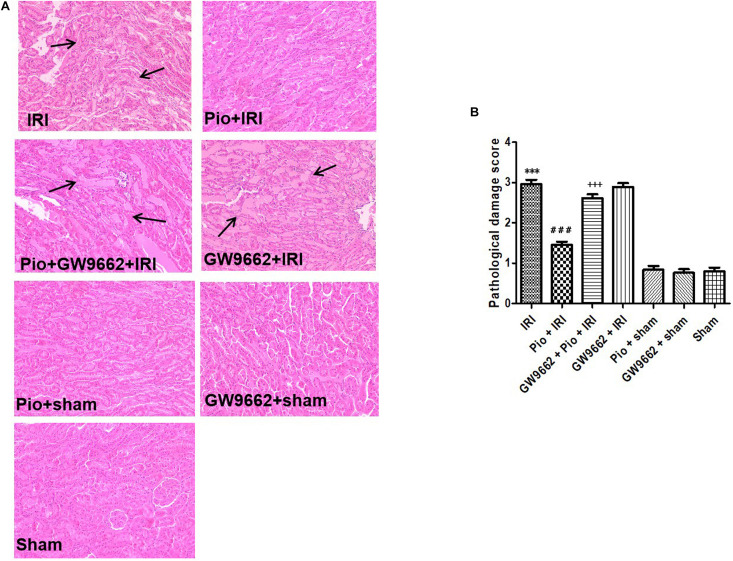
Histological evaluation of the renal tissue at 24 h after renal IRI. **(A)** Representative photographs of kidney sections stained with hematoxylin and eosin (original magnification 200×). **(B)** Histological scoring of the kidney sections. Arrows indicate tubular dilatation, swelling, congestion, vacuolization, and tubular necrosis in the kidneys (*n* = 4). Compared to the sham group, ^∗∗∗^ represents *p* < 0.001; compared to the IRI group, ### represents *p* < 0.001; compared to the Pio + IRI group, +++ represents *p* < 0.001.

The renal tissue sections of rats in the sham operation group exhibited normal tissue morphology. Conversely, the renal tissue samples of rats in the renal IRI group exhibited varying degrees of ischemic injury, which were manifested as renal tubular degeneration, edema, dilation, stasis, vacuolation, and necrosis, thereby resulting in elevated pathological injury scores. The renal IRI rats that were treated with pioglitazone exhibited significant improvement in renal tubular injury, and therefore, they exhibited low pathological injury scores at 24 h postoperatively. However, when used in combination with GW9662, pioglitazone did not alleviate renal tubular injury (Figure 3).

### Pioglitazone Increased PPAR-γ Activity in the Renal Tissue of Rats With Renal IRI

**FIGURE 4:**
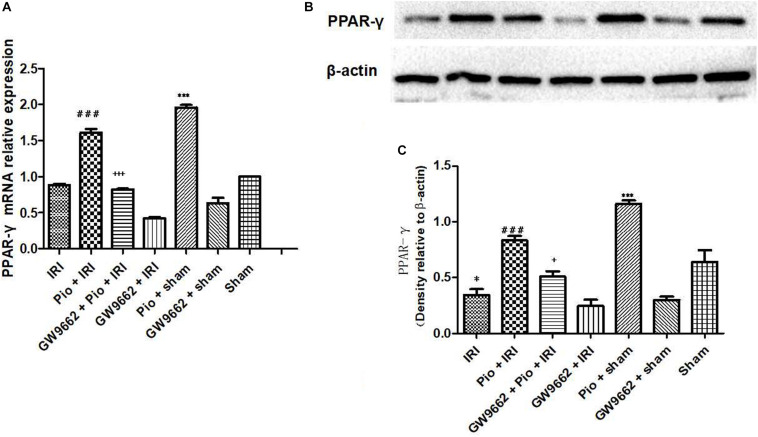
Pioglitazone up-regulates PPAR-γ mRNA and protein expression. **(A)** Quantitative analysis of *PPAR-γ* mRNA in the kidneys at 24 h post renal IRI. **(B)** Representative western blots indicating PPAR-γ protein level in the kidneys at 24 h after renal IRI. **(C)** Quantitative analysis of PPAR-γ protein level in the kidneys. Values are expressed as mean ± SE (*n* = 3). Compared to the sham group, ^∗^ represents *p* < 0.05, and ^∗∗∗^ represents *p* < 0.001; compared to the IRI group, ### represents *p* < 0.001; compared to the Pio + IRI group, + represents *p* < 0.05, and +++ represents *p* < 0.001.

The renal IRI group exhibited significantly lower mRNA and protein expression of PPAR-γ postoperatively than that in the sham operation group. The treatment with pioglitazone significantly up-regulated PPAR-γ mRNA and protein expression in the sham operation group, whereas the administration of pioglitazone in combination with GW9662 did not significantly change the mRNA and protein expression of PPAR-γ in the renal IRI rats. These results indicated that pioglitazone increases the mRNA and protein expression of PPAR-γ in the renal tissue, but its effects are inhibited by GW9662 (Figure 4).

### Pioglitazone Decreased the Expression of NF-κB-Related Proteins (p-IKK-β and IκB-α) in the Renal Tissue of Rats With Renal IRI

**FIGURE 5:**
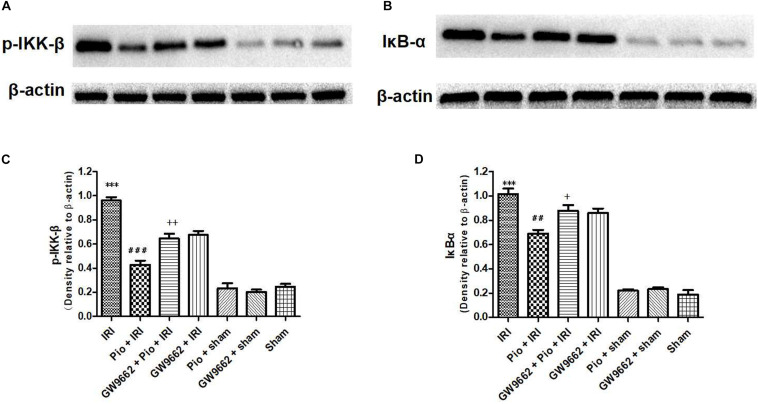
Pioglitazone down-regulates p-IKK-β and IκB-α protein expression at 24 h after renal IRI. **(A,B)** Representative western blots show p-IKK-β and IκB-α protein expression in the kidneys. **(C,D)** Quantitative analysis of p-IKK-β and IκB-α protein expression in the kidneys. Values are expressed as mean ± SE (*n* = 4). Compared to the sham group, ^∗∗∗^ represents *p* < 0.001; compared to the IRI group, ## represents *p* < 0.01, and ### represents *p* < 0.001; compared to the Pio + IRI group, + represents *p* < 0.05, and ++ represents *p* < 0.01.

The renal IRI group exhibited significantly higher p-IKK-β and IκB-α protein expression in the renal tissue than that in the sham operation group. The Pio + renal IRI group exhibited significantly lower expression of p-IKK-β and IκB-α proteins than that in the renal IRI group, whereas the Pio + GW9662 + renal IRI group significantly up-regulated p-IKK-β and IκB-α proteins. These results indicated that GW9662 suppresses the inhibitory effects of pioglitazone on p-IKK-β and IκB-α protein expression (Figure 5).

### Pioglitazone Decreased mRNA Expression of Inflammatory Cytokines, Including TNF-α and MCP-1, in the Renal Tissue of Rats With Renal IRI

**FIGURE 6:**
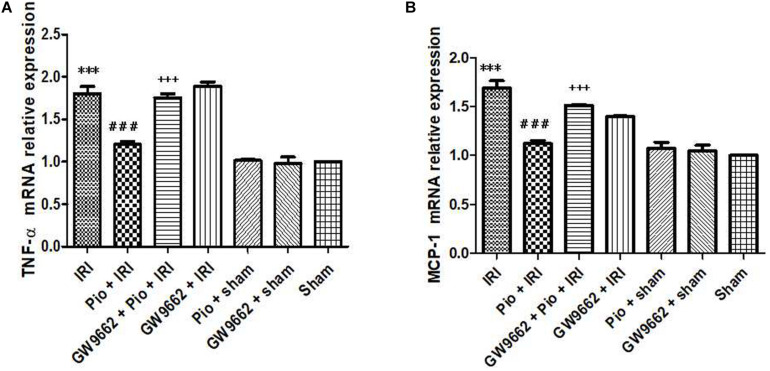
Pioglitazone decreases the mRNA expression of inflammation markers, including TNF-α and MCP-1, in the renal tissues at 24 h after renal IRI. **(A,B)** TNF-α and MCP-1 expression levels in the renal tissue of each group, respectively. Values are expressed as mean ± SE (*n* = 3). Compared to the sham group, ^∗∗∗^ represents *p* < 0.001; compared to the IRI group, ### represents *p* < 0.001; compared to the Pio + IRI group, +++ represents *p* < 0.001.

The mRNA expression of TNF-α and MCP-1 in the renal tissue of the renal IRI group was higher than that in the sham operation group. The Pio + renal IRI group exhibited significantly lower mRNA expression of TNF-α and MCP-1 than that in the renal IRI group. Conversely, the Pio + GW9662 + renal IRI group exhibited significantly higher mRNA expression of TNF-α and MCP-1 than that in the renal IRI group. These findings indicated that GW9662 inhibits pioglitazone-induced downregulation of inflammatory mediators (Figure 6).

## Discussion

We previously demonstrated that pioglitazone ameliorates renal IRI in mice by inhibiting renal cell apoptosis, improving antioxidant activity, and enhancing autophagy (Hu et al., 2012; Zou et al., 2013; Chen et al., 2018). The results of the present study indicate that pioglitazone alleviates the effects of renal IRI in rats by inhibiting the NF-κB signaling pathway and inflammatory response.

In the present study, there was no significant difference in the plasma glucose levels before and 1 h after feeding between the control and pioglitazone treatment groups, respectively. This suggests that the changes in plasma glucose levels are not involved in the renoprotective effects exerted by pioglitazone. Frantz et al. (2005) reported that another antidiabetic treatment, such as acarbose, an oral hypoglycemic agent, could reduce the size of myocardial infarct, and this effect was related to the improvement of postprandial hyperglycemia mediated by acarbose. Therefore, the mechanism by which pioglitazone alleviates renal IRI seems to be different from that of acarbose in cardiac IRI.

Several *in vivo* studies have demonstrated that PPAR-γ agonists can potentially alleviate pathological injury caused due to ischemia-reperfusion. In one such study, the authors used a myocardial IRI model to demonstrate that a PPAR-γ agonist, rosiglitazone, alleviated IRI, reduced infarct and ischemic sizes, and improved ventricular remodeling, thereby improving the recovery of ventricular contractile function and cardiac function (Yue Tl et al., 2001). Other studies have reported that the PPAR-γ agonists alleviate lung and gastrointestinal IRI (Cuzzocrea et al., 2003; Ito et al., 2004; Wada et al., 2004). The protective effects of the PPAR-γ agonists against renal IRI have also been demonstrated (Sivarajah et al., 2003; Chatterjee et al., 2004). In the recent years, our research efforts have been focused on the amelioration of renal IRI using the PPAR-γ agonist, pioglitazone. In this study, pioglitazone was shown to improve the histopathological and biological parameters tested in rats that were subjected to IRI.

Therefore, we investigated the mechanisms underlying the renoprotective effects of pioglitazone. The NF-κB signaling pathway plays a key role in renal IRI-induced renal tubular cell death (Kelly et al., 2003; Meldrum et al., 2003). Renal ischemia induces nuclear translocation and activates the NF-κB pathway in renal tubular cells, thereby promoting ischemia-induced apoptosis. NF-κB overexpression, which is associated with apoptosis, has also been observed in renal tubular cells post renal IRI (Oberbauer et al., 2001). In the present study, we investigated the mechanisms by which pioglitazone ameliorates renal IRI with respect to the NF-κB pathway. Our data indicated that pioglitazone down-regulated the expression of NF-κB-related proteins (p-IKK-β and IκB-α) in the renal tissue of rats with renal IRI. Previous research on IRI in other organs has also demonstrated that pioglitazone ameliorated cerebral and retinal IRI by inhibiting the NF-κB signaling pathway (Zhang et al., 2011, 2013).

NF-κB has been regarded as one of the most important regulatory factors of the inflammatory cascade in renal IRI as it potentially regulates post-IRI cell survival. Various studies have shown that IRI promotes the local or systemic release of pro-inflammatory cytokines, such as TNF-α and IL-6, thereby aggravating organ injury (Wei et al., 2019; Farhadi Moghadam and Fereidoni, 2020)[Bibr B4][Bibr B21]. Therefore, renal IRI can be alleviated by suppressing the renal ischemia-induced inflammatory response (Kasimsetty et al., 2020; Zhang et al., 2020). In a study conducted by Kirpatovskii et al. (2006), the authors demonstrated that renal IRI induces excessive ROS generation through mechanisms, such as increased xanthine oxidase formation, neutrophil respiratory burst, and enhanced catecholamine autoxidation. The generated ROS activates the NF-κB pathway, leading to the up-regulated expression of pro-inflammatory genes and generation of inflammatory mediators. In the present study, we found that pioglitazone reduced the expression levels of the inflammatory cytokines, including TNF-α and MCP-1, in the renal tissue of rats with renal IRI, suggesting that pioglitazone suppressed the renal IRI-induced inflammatory response.

In conclusion, we demonstrated that pioglitazone can potentially ameliorate renal IRI by inhibiting the NF-κB signaling pathway and inflammatory response. Our future research efforts will be focused on further investigating and confirming the effects of pioglitazone to provide experimental data and a theoretical basis for the exploration of novel targets and pathways for the prevention and treatment of renal IRI.

## Data Availability Statement

The raw data supporting the conclusions of this article will be made available by the authors, without undue reservation.

## Ethics Statement

The animal study was reviewed and approved by The Ethics Committee for Animal Experiments of The Second Affiliated Hospital of Nanchang University, China.

## Author Contributions

GZ, ZZ, XX, RH, and HH: acquisition, analysis, and interpretation of data and drafting the manuscript. GZ and HH: study conception and design, analysis and interpretation of data, and drafting the manuscript. All authors contributed to the article and approved the submitted version.

## Conflict of Interest

The authors declare that the research was conducted in the absence of any commercial or financial relationships that could be construed as a potential conflict of interest.
